# The costs of locomotor activity? Maximum body temperatures and the use of torpor during the active season in edible dormice

**DOI:** 10.1007/s00360-017-1080-y

**Published:** 2017-03-21

**Authors:** Claudia Bieber, Jessica S. Cornils, Franz Hoelzl, Sylvain Giroud, Thomas Ruf

**Affiliations:** Department of Integrative Biology and Evolution, Research Institute of Wildlife Ecology, University of Veterinary Medicine Vienna, Savoyenstraße 1, 1160 Vienna, Austria

**Keywords:** Arboreal, Beech mast, Small mammal, Foraging, Date of parturition

## Abstract

Measuring *T*_b_ during the active season can provide information about the timing of reproduction and the use of short bouts of torpor and may be used as a proxy for the locomotor activity of animals (i.e., maximum *T*_b_). This kind of information is especially important to understand life-history strategies and energetic costs and demands in hibernating mammals. We investigated *T*_b_ throughout the active season in edible dormice (*Glis glis*), since they (*i*) have an expensive arboreal life-style, (*ii*) are known to show short bouts of torpor, and (*iii*) are adapted to pulsed resources (mast of beech trees). We show here for the first time that maximum *T*_b_’s in free-living active dormice (during the night) increase regularly and for up to 8 h above 40 °C, which corresponds to slight hyperthermia, probably due to locomotor activity. The highest weekly mean maximum *T*_b_ was recorded 1 week prior to hibernation (40.45 ± 0.07 °C). At the beginning of the active season and immediately prior to hibernation, the mean maximum *T*_b_’s were lower. The time dormice spent at *T*_b_ above 40 °C varied between sexes, depending on mast conditions. The date of parturition could be determined by a sudden increase in mean *T*_b_ (plus 0.49 ± 0.04 °C). The occurrence of short torpor bouts (<24 h) was strongly affected by the mast situation with much higher torpor frequencies in mast-failure years. Our data suggest that locomotor activity is strongly affected by environmental conditions, and that sexes respond differently to these changes.

## Introduction

While the variation of body temperature (*T*_b_) during hibernation has been intensively investigated in many species (reviewed in Ruf and Geiser 2015), our knowledge of *T*_b_ patterns in hibernators during the active season is still sparse. Some studies have focused, however, on *T*_b_ prior to the onset of hibernation to investigate whether the hypothalamic *T*_b_ set point is already adjusted during the active season in preparation for the upcoming hibernation season. Indeed, a decline in *T*_b_ starts already several weeks prior to hibernation in several species (e.g., 45 days in the arctic ground squirrel (Sheriff et al. 2012), 14 days in the Alpine marmot (Arnold et al. 2011), and 5–16 days in Syrian hamsters (Arai et al. 2005; Chayama et al. 2016). This pre-adjustment seems to be in line with the view that several physiological changes, like adjustments in appetite, organ size, and body mass occur throughout the active season in preparation for hibernation (Dark 2005; Florant and Healy 2012; Hume et al. 2002). However, beyond this topic, fluctuations in *T*_b_ during the active season, especially in free-living hibernators, received only little attention.

It has been shown that the measurement of *T*_b_ during the active season can provide valuable information about phenological patterns in free-living Arctic ground squirrels (Williams et al. 2011). For example, the date of parturition can be estimated with high accuracy based on an abrupt increase in mean *T*_b_ of gestating females. Further, daily rhythmicity in *T*_b_ measurements allows to differentiate between time spent euthermic in hibernacula (prior to and after hibernation) and actual hibernation duration (Williams et al. 2011). This kind of information is especially important to understand life-history strategies as well as energetic costs and demands in hibernating mammals.

Measuring *T*_b_ during the active season can also provide information on locomotor activity of animals. It is well known that physical exercise leads to an increase in *T*_b_ in several species, ranging from small mammals to humans (Hart and Heroux 1955; Reilly and Brooks 1986). Not surprisingly then, there is a close correlation between changes in *T*_b_ and locomotor activity (Heldmaier et al. 1989; Fuller et al. 1998; Weinert and Waterhouse 1998; Hart and Heroux 1955; Golombek et al. 1993). However, the energetic costs of different styles of locomotion vary extremely and are likely to increase maximum *T*_b_ during the daily active phase differently. In particular, in arboreal mammals, the increase in metabolic rate during the active phase is more pronounced than in terrestrial mammals (Karasov 1992). In the case of the arboreal Leadbeater’s possum, the mean metabolic rate during the active phase is 9.1 times that of the basal metabolic rate (BMR, Smith et al. 1982, reviewed in; Karasov 1992). These extreme metabolic rates are expected to lead to elevated maximum *T*_b_ during the active phase.

While maximum *T*_b_ can provide some information about the locomotor activity of the animals, minimum *T*_b_, on the other hand, indicates phases of torpor during the active season. Some hibernating species also show short phases of torpor (typically <24 h) during the active season (e.g., Sheriff et al. 2012; Johansen and Krog 1959; Geiser 1987; Webb and Skinner 1996; Turbill et al. 2003). These short bouts of torpor are more likely to occur under conditions of reduced food availability or low ambient temperature (*T*_a_) during the daily resting phase. The significant reduction of daily energy expenditure through the use of short torpor bouts in summer allows hibernators, for instance, to allocate more energy to growth or pre-hibernation fattening (Giroud et al. 2014).

For the study of maximum and minimum *T*_b_ throughout the active season, the edible dormouse (*Glis glis*, henceforth ‘dormouse’) is an interesting model species for several reasons. First, dormice have an arboreal life-style and climb easily between shrub level, or even ground level, up into the canopy of trees at about 30–45 m height (Müller 1988). This is also reflected by the diet of dormice, which contains both seed buds and seeds of trees (oak, *Quercus spec*. and beech, *Fagus sylvatica*) from up in the canopy and raspberries (*Rubus idaeus*) or blackberries (*Rubus fruticosus*), which grow close to the ground (Sailer and Fietz 2009). Second, dormice are fat-storing hibernators. Thus, they have to carry large body fat reserves, especially just prior to hibernation, which should be particularly costly for an arboreal species. Third, their major food source in European woods, the seeds of beech, are pulsed resources with extreme fluctuations in abundance between years (Ostfeld and Keesing 2000). While in the so-called mast years the seed abundance is overwhelming, a total lack of seed production on a large scale can be observed in non-mast years. Dormice are strongly adapted to the mast-pattern of beeches and reproduce in central Europe only in years when this food source is abundant (Bieber 1998; Schlund et al. 2002; Ruf et al. 2006). While dormice already feed on inflorescences and seed buds in spring, the highest proportions of seeds are consumed late in the active season (Juškaitis et al. 2015; Sailer and Fietz 2009). Fourth, dormice are known to exhibit short bouts of torpor during the active season (Wilz and Heldmaier 2000; Hoelzl et al. 2015) and thus allow investigating the use of torpor in combination with an energy demanding arboreal life-style.

Since dormice need to accumulate body fat reserves prior to winter, we expected that the investment into foraging and, hence, the associated increases in *T*_b_ during nocturnal activity should be especially pronounced in this species. If so, then maximum *T*_b_ may represent a proxy for investment in locomotion, as it will reflect the actual workload and associated thermogenesis caused by muscular work. We hypothesized that both maximum daily *T*_b_’s (i.e., locomotor activity) and minimum *T*_b_’s (i.e., use of torpor) will (*i*) change throughout the active season, (*ii*) be affected by fluctuations in environmental conditions, such as yearly differences in food supply, and (*iii*) are affected by reproductive behavior.

## Materials and methods

### Animals and study sites

We analyzed *T*_b_ records from 77 dormice implanted with temperature loggers (details see below). Ten of these dormice were recorded twice, in two consecutive years (2005 and 2006). The 87 datasets were recorded between 2005 and 2014, (2005 *n* = 14, 2006 *n* = 17, 2008 *n* = 18, 2012 *n* = 12, 2013 *n* = 12, 2014 *n* = 11). From 2005 to 2008, the implanted animals were held in large outdoor enclosures (6 × 4 × 3.5 m each) in Vienna, Austria (362 m a.s.l., 48°13′N,16°16′E) and were fed ad libitum with rodent chow (Altromin 1314, FORTI). In 2008, dormice were captured at our field study site in the Vienna woods (St. Corona am Schöpfl, Austria, about 40 km from the enclosures in Vienna) during summer/fall and were transported, implanted, and kept in enclosures until the end of the hibernation season (early summer 2009). They were released at the capture point after explantation of the data loggers. Between 2012 and 2014, *T*_b_ data were recorded year-round in free-living dormice at our study site in the Vienna woods. All dormice were marked individually (PIT tags, 13.8 × 2.1 mm; Virbac, BackHomeBioTec, Bad Oldesloe, Germany). We distinguished between the age classes yearling (after first hibernation) and adult (beyond second hibernation) based on fur color and body mass (for details see Bieber 1998). At the field study site, nest-boxes were controlled at fortnight-intervals to check whether females had given birth. The date of parturition was estimated based on size and development of the juveniles (Koenig 1960; Bieber and Ruf 2004). For further details on the study sites and housing conditions of dormice, see Bieber and Ruf (2009), Bieber et al. (2014), and Hoelzl et al. (2015).

*T*_a_ was recorded with data loggers (DS1922L, Dallas/Maxim, Maxim Integrated, San Jose, CA, US; Resolution 0.5 °C, calibrated in a water bath) at 2 m height in a shaded location close to the enclosures and at the field study site, respectively. The beech (*Fagus sylvatica*) mast situation was determined via visual observation at the field study site. In 2012 and 2014, we observed a total lack of beech seeds at the whole study site (mast-failure years); 2013 was a full mast year with a large amount of seeds available.

### Implantation

All dormice were implanted with wax-coated iButtons (DS1922L, Dallas/Maxim, Maxim Integrated, San Jose, CA, US; Resolution 0.5 °C, calibrated in a water bath prior to implantation) at the Research Institute of Wildlife Ecology (FIWI), University of Veterinary Medicine Vienna. The iButtons were implanted either subcutaneously (in the lateral area of the thorax, caudal of the scapula; 2005–2006) or intraperitoneally (due to changes in the animal ethics guidelines). Anesthesia for implantations was induced by a subcutaneous injection of 50 mg/kg Ketamine (Ketamidor^®^ 10%, Richter Pharma Wels, Austria) and 8 mg/kg Xylazine (Rompun^®^ 2%, Bayer, Leverkusen, Germany) and maintained by 1.5% isoflurane in an oxygen stream via a facemask. Post-surgical analgesia (5 mg/kg Ketoprofen, subcutaneously) was provided. For the detailed description of the implantation procedure, see Bieber and Ruf (2009), Bieber et al. (2014), and Hoelzl et al. (2015). After implantation, the animals recovered individually in cages (59 cm x 49 cm x 41 cm), provided with shelter, food, and water ad libitum, for 5–7 days. Implantations were carried out between May and September. From 2012 to 2014, animals were captured in nest-boxes at the field study site and transported to the laboratory. The nest-boxes were closed to prevent other dormice from moving in while the animals were absent. After implantation and healing of the wound (5–7 days), animals were released into the nest-box where they had been captured. Some sunflower-seeds (approx. 20 g) were provided into the nest-box to facilitate the release. Explantation followed the same procedure as implantation and was carried out about 1 year after implantation.

### Statistics

*T*_b_ was recorded at approximately hourly intervals (3850 s) to cover 1 year of data recording. Since iButtons were implanted during the active season, we obtained *T*_b_ data from 3 to 21 weeks (mean 8.6 ± 0.52 weeks, in total 751 weeks) prior to the onset of hibernation, depending on the date of implantation. Onset of hibernation was determined as the date of entrance into the first multiday torpor bout in fall. Body mass data could not be included in this analysis since we did not recapture the implanted dormice at regular intervals throughout the active season.

For better comparison with literature data (e.g., Sheriff et al. 2012), we calculated and show maximum daily *T*_b_ in weekly means prior to hibernation (counting weeks backward starting from 0 = onset of hibernation). For further evaluations, however, we determined whether the predictor ‘week of year’ (starting with the first week in January as 1) described our *T*_b_ data better than ‘week prior to hibernation.’ We therefore used mean *T*_b_ as response variable, as this variable encompasses both maximum *T*_b_ and minimum *T*_b_, and calculated the full models (mean *T*_b_~age + sex + mean *T*_a_) for both weekly variables. Because ‘week of year’ resulted in a larger fraction of variance explained, we decided to use week of year as a predictor for all further modeling procedures (free-living dormice: R^2^ ‘week of year’ = 0.31 and R^2^ ‘week prior to hibernation’ = 0.22; all dormice: R^2^ ‘week of year’ = 0.38 and R^2^ ‘week prior to hibernation’ = 0.31).

As a proxy for the daily time spent highly active, we computed the daily time spent slightly hyperthermic, i.e., hours *T*_b_ > 40 °C/days (always occurring during night, the typical activity phase in dormice). This assumes that, as in other small mammals (Hart and Heroux 1955), mild hyperthermia in dormice can be caused by elevated locomotor activity, most likely due to mating behavior or intensive foraging. To correct for the potential effects of *T*_a_ on *T*_b_ (Hart and Heroux 1955; Humphries and Careau 2011), we included mean *T*_a_ in all models analyzing maximum *T*_b_, mean *T*_b_, or the daily time spent at *T*_b_ > 40 °C.

Torpor was defined as a *T*_b_ < 32 °C and calculated as days with torpor bouts per week. Short periods of torpor occurred always during daytime (Hoelzl et al. 2015). To adjust for possible effects of *T*_a_ on torpor proneness, we included daily minimum *T*_a_ in models evaluating torpor frequency.

The highest minimum *T*_b_ in females was compared between reproductive (2013) and non-reproductive (2012, 2014) females, using a linear model. Always the highest value per individual during week 30–35 (lactation period) was analyzed.

Data were analyzed with R, version 3.2.4 (R Core Team 2016) using the package nlme (Pinheiro et al. 2016) to compute linear mixed effect (lme) models (full models are shown in Table 1). Since individuals were repeatedly measured, we used animal ID as a random effect in our modeling procedure. All models were corrected for autocorrelation of residuals using first-order autoregressive models (package nlme) and results are given as Anova tables with type 3 sums of squares (package car, Fox and Weisberg 2011). Because of non-normally distributed residuals, the response variables maximum *T*_b_ and mean *T*_b_ were Box-Cox transformed using package car (Fox and Weisberg 2011). All possible interaction terms were tested, and only significant interactions were included in the final model. All means are given ± SEM (standard error of the mean).

**Table 1:** Anova tables for lme models; (a) Maximum *T*_b_ in free-living dormice (only values >35 °C were considered to exclude torpor from the analysis), (b) Activity (h/days at *T*_b_ > 40 °C) in free-living dormice, (c) Frequency of torpor bouts in free-living dormice, mast = mast year (2013) compared with 2 mast-failure years (2012, 2014), (d) Site effects (free-living versus enclosure) on activity patterns, and (e) Site effects on torpor patterns

Predictor	*χ* ^2^	*df*	*P-*value
(a) Maximum *T*_b_, free-living dormice			
Week prior to hibernation	117.95	20	<0.0001***
Sex	3.22	1	0.0727
Age	1.11	1	0.2922
Mast	7.39	1	0.0066**
*T*_a_ mean	9.16	1	0.0025**
(b) h/days at *T*_b_ > 40 °C, free-living dormice			
Week of year	177.92	21	<0.0001***
Sex	4.30	1	0.0381*
Age	1.04	1	0.3073
Mast	17.67	1	<0.0001***
*T*_a_ mean	5.58	1	0.0182*
Sex × food	8.59	1	0.0034**
(c) Torpor bouts/week, free-living dormice			
Week of year	104.22	21	<0.0001***
Sex	0.53	1	0.4648
Age	0.21	1	0.6431
Mast	46.91	1	<0.0001***
*T*_a_ min	28.82	1	<0.0001***
(d) h/days at *T*_b_ > 40 °C, all dormice			
Week of year	129.18	23	<0.0001***
Sex	1.17	1	0.2789
Age	0.0056	1	0.9401
Site	18.68	1	<0.0001***
*T*_a_ mean	10.15	1	0.0014**
(e) Torpor bouts/week, all dormice			
Week of year	131.44	23	<0.0001***
Sex	0.13	1	0.7235
Age	0.35	1	0.5534
Site	2.50	1	0.1141
*T*_a_ min	13.01	1	0.0003***

Significance levels are indicated with <0.05 = *, <0.05 >0.001 = **, <0.001 = ***

While we recorded core *T*_b_’s in free-living dormice (2012–2014), we measured subcutaneous *T*_b_’s in enclosure held animals (2005–2006). To allow the comparison between free-living and enclosure-housed dormice, we used data recorded in 2008, when we measured intraperitoneally implanted animals in our enclosures. In the same environment (enclosures), the maximum *T*_b_ measured intraperitoneally was on average 1.8 °C higher than the maximum T_b_ measured subcutaneously. Since the resolution of the iButtons is 0.5 °C, we corrected all subcutaneously obtained data by decreasing the corresponding threshold of activity in this dataset to 38 °C (−2 °C). In the following we refer, however, always to the threshold of 40 °C, presuming that this is the more accurate description of the real core temperature.

## Results

### Maximum T_b_ in free-living dormice

**Fig. 1:**
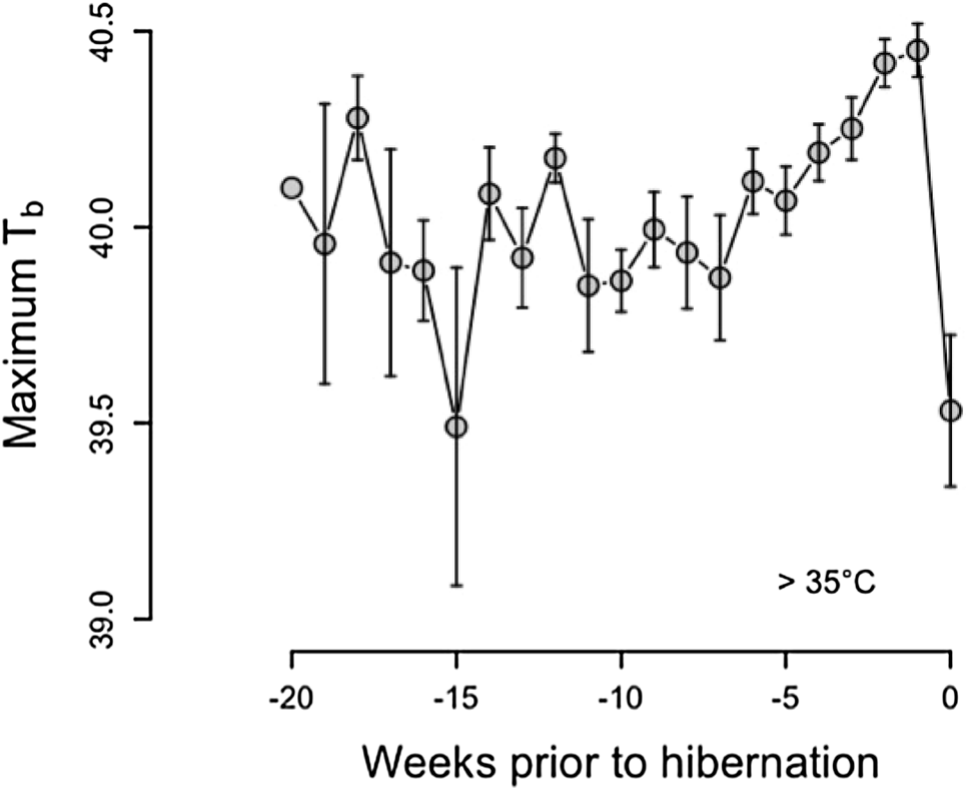
Maximum body temperature in edible dormice prior to hibernation. 0 = onset of hibernation; negative weeks are the weeks counted backwards from the onset of hibernation. To avoid effects of multiday torpor (which occurred for few days in three cases) during the active season, only maximum *T*_b_’s > 35 °C were considered. Given are weekly means ± SEM

Focusing on free-living dormice, we observed that maximum *T*_b_ was significantly affected by week prior to hibernation, mast, and mean *T*_a_ (Table 1). Maximum *T*_b_’s were higher in a mast year and increased with increasing *T*_a_. Since we included *T*_a_ into our modeling procedure, we corrected all other variables for temperature effects (see also all other models, Table 1). Age and sex did not significantly affect maximum *T*_b_. Maximum *T*_b_ increased strongly between week −7 and −1 prior to hibernation (Fig. 1). The highest weekly mean maximum *T*_b_ was recorded in week −1 (40.45 ± 0.07 °C). Only in the last week prior to hibernation (week 0), the maximum T_b_ decreased (39.53 ± 0.19 °C). In weeks −20 to −7, we observed strong fluctuations between the weekly means of maximum *T*_b_ in dormice (Fig. 1).

### Activity and reproduction in free-living dormice

**Fig. 2:**
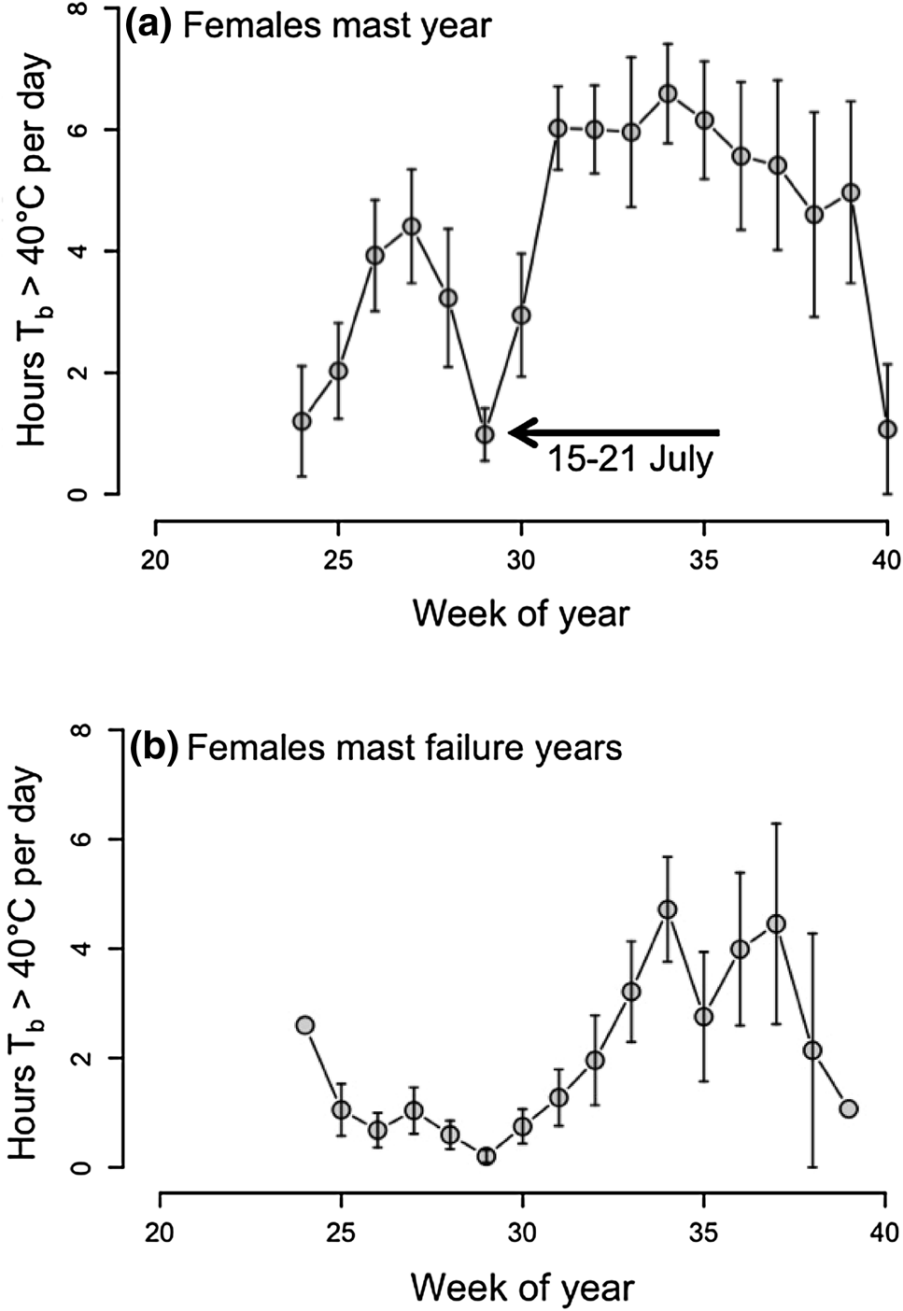
Hours per day spent at *T*_b_ > 40 °C in free-living female dormice shown as weekly means ± SEM, **a **females during a mast year (2013), **b** females during mast-failure years (2012, 2014). There was a significant interaction between sex and age. Week of year 20 = mid May, week of year 40 = end of September/beginning of October). Females were more active in the mast year. Further, a decline in body temperature in week 29 (15–21 July) could only be observed in females prior to parturition in a mast year (compare Fig. 4)

**Fig. 3:**
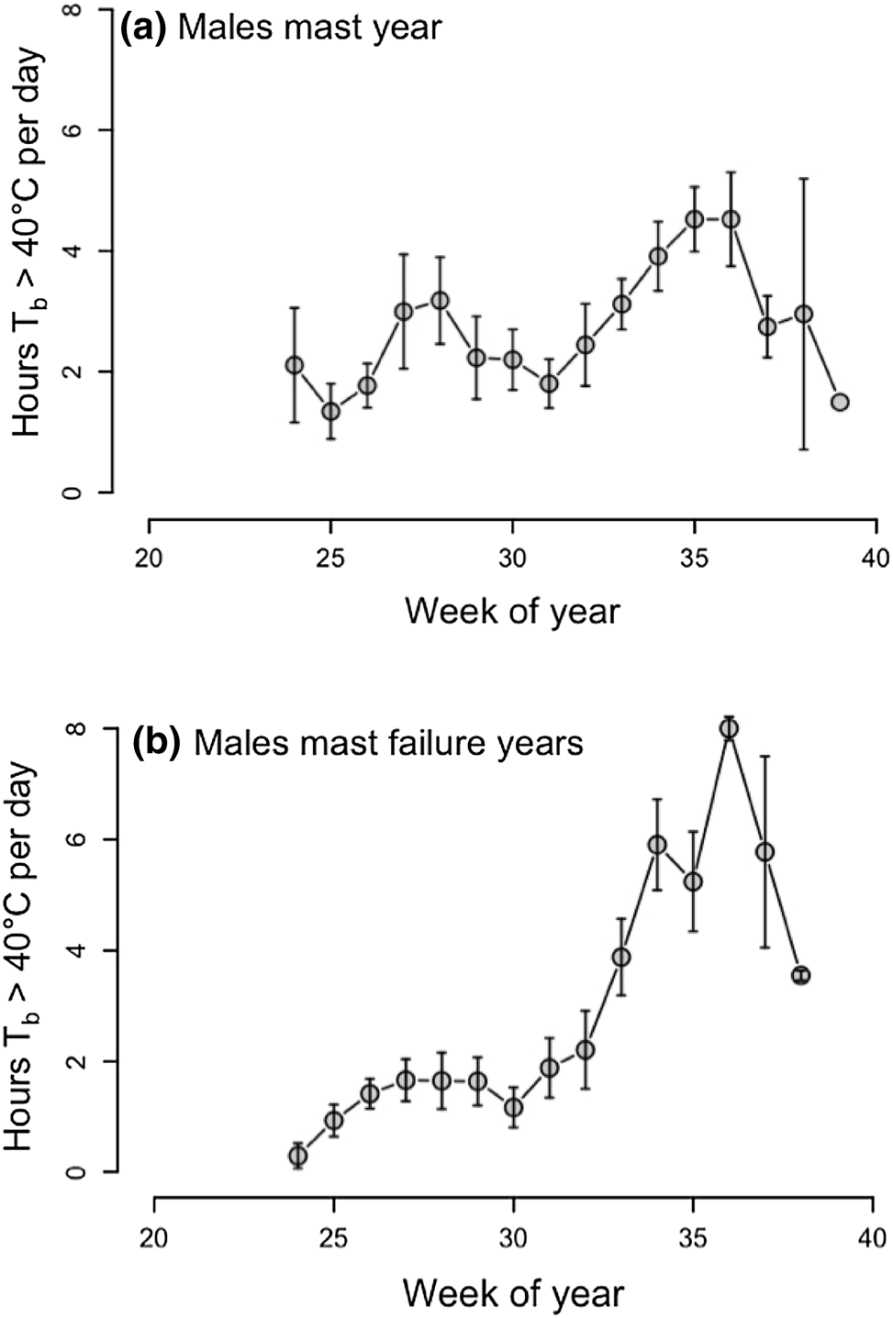
Hours per day spent at *T*_b_ > 40 °C in free-living male dormice weekly means ± SEM, **a** males during a mast year (2013), **b** males during mast-failure years (2012, 2014). Week of year 20 = mid May, week of year 40 = end of September/beginning of October. There was a significant interaction between sex and age. In mast-failure years, males showed an even higher activity towards the end of the active season. The largest amount of time spent at *T*_b_ > 40 °C was observed in week 36 (end of August) in males in a mast-failure year

Weekly means of time spent at *T*_b_ > 40 °C/days, i.e., hours spent hyperthermic during the active phase at night, were significantly affected by the week of year, sex, *T*_a_, the mast situation, and the interaction between sex and mast (Table 1). This significant interaction was due to much higher weekly means of h *T*_b_ > 40 °C/days in females in a mast year (mast year: 4.20 ± 0.29 h, mast-failure year: 2.06 ± 0.26 h; Fig. 2), but only a small difference in males (males: mast year: 2.53 ± 0.16 h, mast-failure year: 2.55 ± 0.21 h). However, males seemed very active (up to 8 h *T*_b_ > 40 °C/days during night-time) especially towards the end of the active season in mast-failure years (Fig. 3). Independent of the mast situation, all dormice showed the highest values of time spent at *T*_b_ > 40 °C/days around week 35 (the last week of August, Figs. 2, 3). Time spent at these high *T*_b_’s increased significantly with increasing mean *T*_a_.

**Fig. 4:**
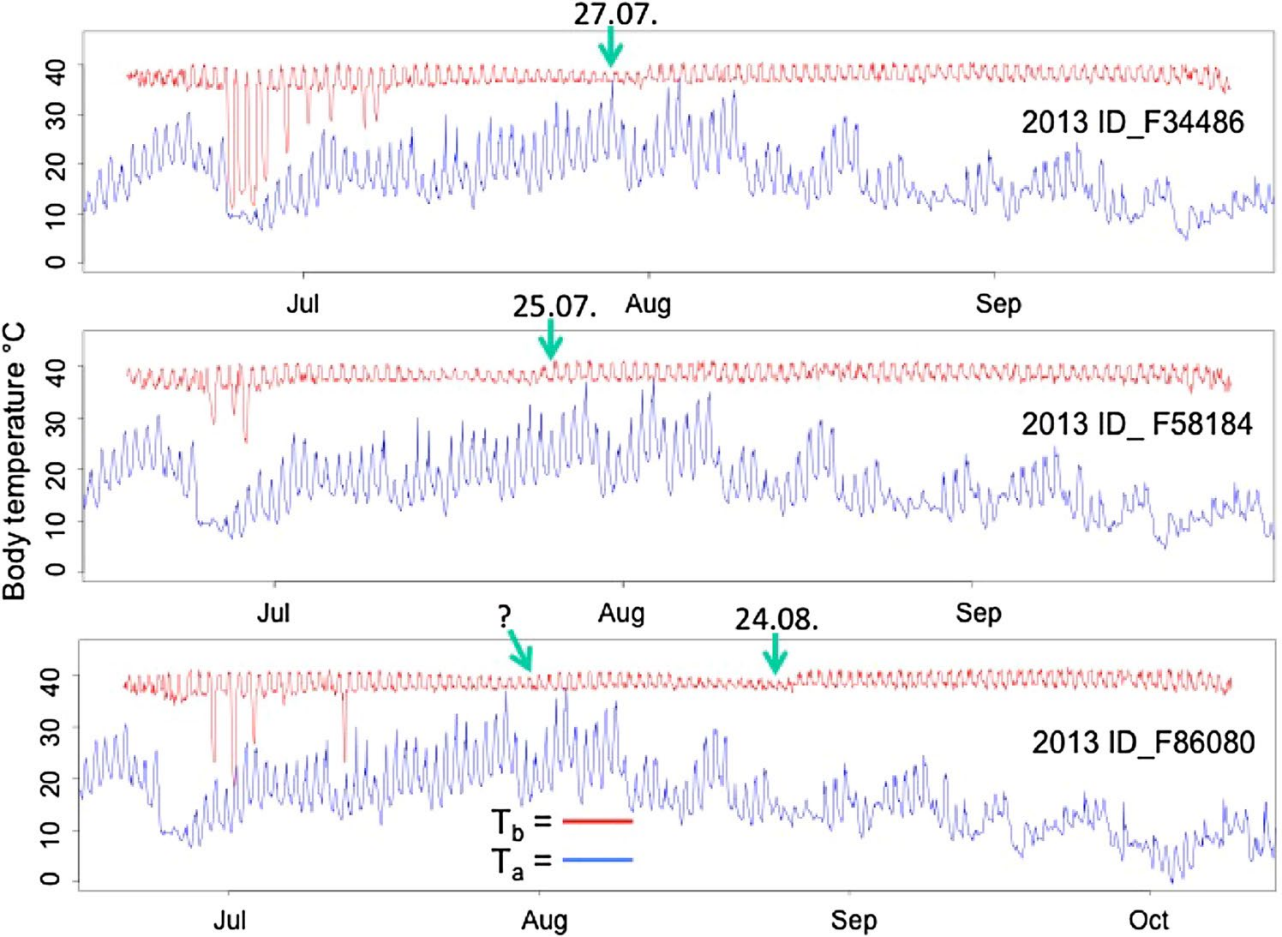
Raw data of *T*_b_ records for three exemplary free-living females in 2013 (mast year) together with *T*_a_ (air temperature 2 m above ground). The *green arrows* indicate the calculated birth date (the age of juveniles was estimated by development and size). They approximately matched the time of the lowest maximum *T*_b_. Female F 86080 was observed with only one litter matching the second decline in maximum *T*_b_. The litter was born very late in the year (Aug 26). Based on temperature records, we assume that she might have lost a first litter shortly after parturition (~Jul 30 indicated by a question mark,* lower panel*) (Color figure online)

Due to nest-box controls we observed in the mast year all implanted females (*n* = 7) with a litter and could estimate the birth date of juveniles (see “Material and methods” section) for 6 litters. The estimated birth date varied by ±3 days around the dates with a sudden increase in daily mean *T*_b_ (plus 0.49 ± 0.04 °C, Fig. 4), most likely indicating the day of parturition.

The average time spent at *T*_b_ > 40 °C/days decreased to a minimum of <2 h/days in week 29 (Fig. 2a) a time when dormice were pregnant (three examples are shown in Fig. 4). However, minimum *T*_b_ in females stayed fairly constant between weeks 28–30 (week 28: 36.87 ± 0.13 °C, week 29: 36.96 ± 0.13 °C, week 30: 36.91 ± 0.15 °C, end of July, beginning of August), when time spent at *T*_b_ > 40 °C/days was lowest. Thus, the observed decrease in the time spent at *T*_b_ above 40 °C/days did not affect the lower boundary of *T*_b_ in pregnant females (compare Figs. 2a, 4). During time of lactation (week 30–35), the highest minimum *T*_b_, which is likely to reflect *T*_b_ at resting metabolic rate (RMR), was 1.37 °C higher in reproductive females (all females in 2013, 37.43 ± 0.15 °C) than in non-reproductive females (all females in 2012 and 2014, non-mast years, 36.06 ± 0.30 °C). This difference was significant (F_1,13_ = 15.42, *P* = 0.0017).

### Short torpor in free-living dormice

**Fig. 5:**
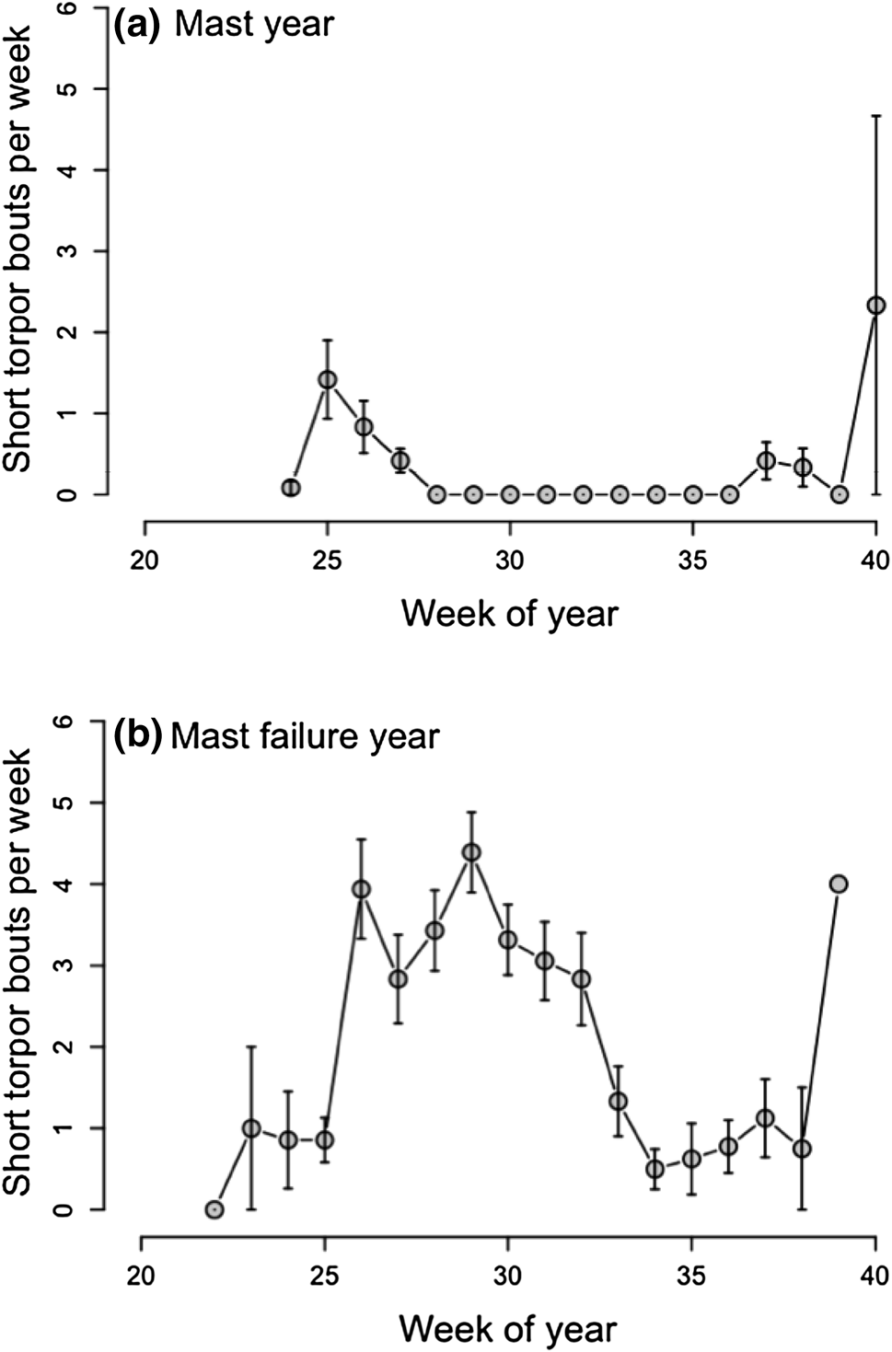
Mean number ± SEM of short torpor bouts per week, **a** in a mast year, **b** in a mast-failure year in free-living dormice. Week of year 20 = mid May, week of year 40 = end of September/beginning of October. Torpor occurred frequently during summer (June–August) only in the mast-failure years (2012 and 2014). Dormice did not show torpor during gestation and lactation in a mast year (2013)

**Fig. 6:**
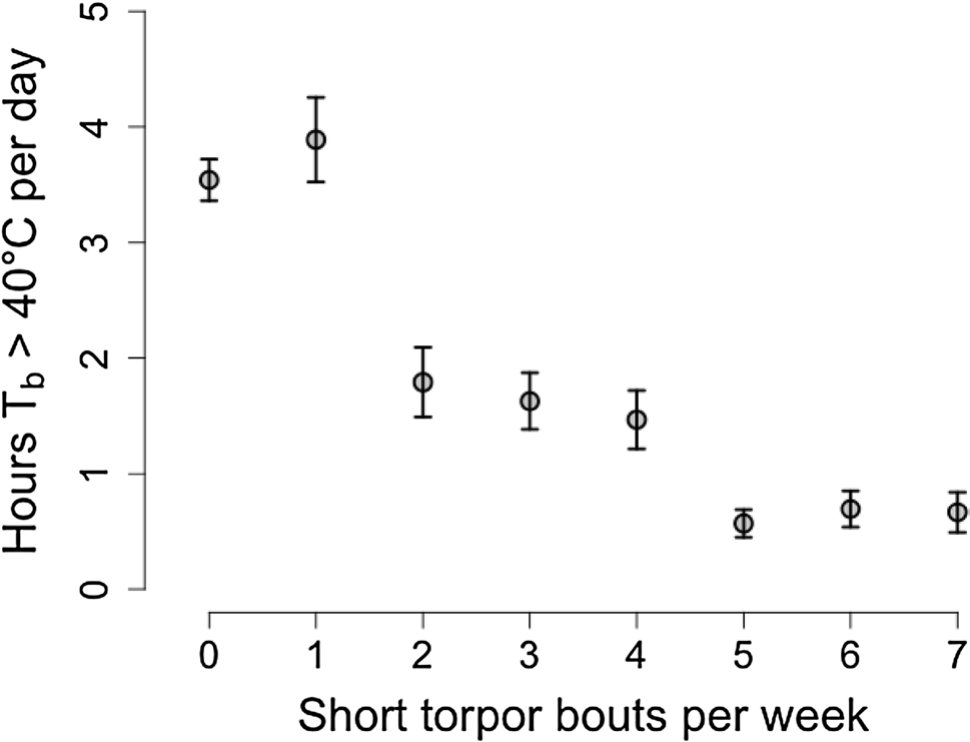
Correlation between weekly means of hours of time spent in slight hyperthermia (h/days *T*_b_ > 40 °C) per day and frequency of short torpor bouts during the active season in free-ranging dormice (correlation coefficient = −0.39, *P* < 0.0001)

The frequency of torpor was affected by the week of year, minimum *T*_a,_ and the mast situation (Table 1). We found no significant interaction in this model. Whereas short torpor was virtually absent in mast years (Fig. 5a), it occurred frequently in mast-failure years (mast year: 0.26 ± 0.06 short torpor bouts/week, mast-failure year: 2.29 ± 0.15 short torpor bouts/week). In mast-failure years, the highest frequency of short torpor bouts occurred during summer (week 26–32, June–August) but not immediately prior to hibernation (Fig. 5b). The frequency of short torpor bouts/week showed a significant negative correlation with the time spent hyperthermic during the night (weekly means of h *T*_b_ > 40 °C/days) in free-living animals (*t* = −8.67, *df* = 430, *P* < 0.0001, correlation coefficient = −0.39, Fig. 6). Indeed, the lowest frequency of short torpor occurred simultaneously with the highest values of time spent at slight hyperthermia around week 35 (last week in August, Figs. 2, 3, 5).

### T_b_ in free-living versus enclosure-housed dormice

To validate that the time spent at *T*_b_ > 40 °C/days reflected phases of intensive locomotor activity in dormice, we compared data from free-living dormice with enclosure-housed animals. The weekly means of time spent at *T*_b_ > 40 °C/days was significantly affected by week of year, mean *T*_a_, and site (see Table 1). As expected, the ad libitum fed animals in the enclosures were significantly less active (field: 2.90 ± 0.13 h, enclosure: 2.32 ± 0.14 h). The torpor frequency, however, was only affected by week of year and minimum *T*_a_, but not by site (field: 1.33 ± 0.10 short torpor bouts/week, enclosure: 1.71 ± 0.14 short torpor bouts/week, Table 1). In both models, there were no significant interactions.

## Discussion

We show here for the first time that maximum *T*_b_’s in active dormice (during the night) increase regularly and for up to 8 h above 40 °C, which corresponds to slight hyperthermia, throughout the active season. Further, the time they spent at these temperature levels varied between sexes and mast conditions. Although it was already documented that dormice reach maximum *T*_b_’s up to 41 °C during the active season (Hoelzl et al. 2015), the amount of time spent at slight hyperthermia per day has never been investigated. While it is known that *T*_b_ can reach values above 40 °C during activity in small mammals living under heat load in deserts or the tropic (e.g., Chappell and Bartholomew 1981; Bondarenco et al. 2014), this has never been observed in small mammals inhabiting forests in temperate zones.

### T_b_ prior to hibernation

Interestingly, we observed a strong and continuous increase in maximum *T*_b_ starting 6 weeks prior to the onset of hibernation (Fig. 1). Only in the last week, when the frequency of short torpor increased, maximum *T*_b_ decreased strongly. These results conflict with other studies, in which free-living hibernators decreased mean *T*_b_ (Alpine marmot) or even maximum *T*_b_ (Arctic ground squirrel) 2–6 weeks prior to hibernation (Sheriff et al. 2012; Arnold et al. 2011). However, a major difference between the investigated species is that ground squirrels and marmots are terrestrial, whereas dormice are arboreal. Thus, it is likely that the mode of locomotor activity (terrestrial versus arboreal) plays an important role in determining *T*_b_’s prior to hibernation. Investigating arboreal hibernators, like some dormice species or small lemurs therefore likely will increase our understanding of temperature regulation and nocturnal activity during the active season and prior to hibernation.

### Patterns of hours spent at T_b_ > 40 °C/days in free-living dormice

We observed that the time spent at *T*_b_ > 40 °C/days was affected by an interaction between sex and mast. While *T*_b_ in males stayed on average ~2.5 h/days above 40 °C, independent of the mast situation, females more than doubled their time spent slightly hyperthermic in a mast year compared to a mast-failure year (~4 to ~2 h/days). These high *T*_b_ levels and therefore most likely high energetic costs in females were related to the lactation period, which only occurred in a mast years (all implanted females were observed with a litter). In mast-failure years, however, there was a total lack of reproduction. Our results give therefore a hint for differences in costs of reproduction between sexes, with much higher costs for females. This raises the question which part of the increased time spent at *T*_b_ > 40 °C in lactating females was related to increased locomotor activity or increased basal metabolic rate (BMR). Indeed an impact of lactation on *T*_b_ is expected by the heat dissipation limit theory (Speakman and Król 2010a). We can show here that during time of peak lactation the highest values of minimum *T*_b_, (days without torpor), which is likely to reflect *T*_b_ at RMR, was significantly higher (1.37 °C) in reproductive females than in non-reproductive females. Thus, a slight elevation of *T*_b_ was probably caused by an increase of BMR during lactation. However, the major part of hyperthermia with *T*_b_ increasing another 3 °C (to >40 °C) was apparently caused by intensive locomotor activity during lactation. For another arboreal mammal, Leadbeater’s Possum, it was shown that the animals generally entered and left their nests as often as 50 times throughout their 10.3 h nightly activity period, which was related to extremely high energy expenditures in this species (Smith et al. 1982). It is likely that female dormice, which raise their young alone, return to their litters (in our case nest-boxes in about 2–3 m height, positioned at a tree) several times per night to lactate or provide food and may therefore show high levels of locomotor activity and energy expenditure. Under laboratory conditions, dormice reach average daily metabolic rates, i.e., including metabolism during activity, as high as 6.9 x BMR during peak lactation, and assimilate significantly more energy than non-reproductive females (Zoufal 2005). We expect that the costs of lactation in free-living dormice are even higher, since locomotor activity in animals fed ad libitum in the laboratory study was certainly minimal.

Both sexes, however, showed a slight increase in the time spent at *T*_b_ > 40 °C/days (from ~2 to ~4 h), at the beginning of the active season (weeks 25–28, end of June–beginning of July, about 1 month prior to parturition, Fig. 4) which might be related to mating behavior in the mast year. Indeed this increase could not be observed in mast-failure years, when dormice almost never spent more than 2 h at *T*_b_ > 40 °C/days (Figs. 2, 3). It is well known that dormice do not invest into reproduction in mast-failure years and that males do not even develop competent testes in these years (e.g., Bieber 1998; Fietz et al. 2004; Joy et al. 1980). Body mass is at a minimum in both sexes during the mating season in mast years (Bieber 1998). Thus, it is likely that the observed increased time spent at *T*_b_ > 40 °C/days during the mating season was not caused by an increase in foraging alone but was also due to mating.

Independent of the year (i.e., the mast situation) or sex, we observed an increase in the time spent at *T*_b_ > 40 °C towards the onset of hibernation. An increase in phases of slight hyperthermia might reflect locomotor activity, most likely related to an increase in food availability and the need to fatten up prior to hibernation during this time period. In mast years, when beech seeds were overabundant, an increase in locomotor activity (i.e., foraging) might also be related to an increased energy demand due to additional cost of reproduction. In mast-failure years, on the other hand, the increased search for less energy-rich food may be the major reason for an increased locomotor activity. Indeed, the diet of dormice is known to change between years with a shift towards less energy-rich food or other seeds in mast-failure years (Juškaitis et al. 2015; Fietz et al. 2005). Thus, it is possible that the animals invested more time in foraging during these years, although the demands were lower because of the skipped reproduction (in females more than in males, see above). We cannot rule out, however, that the heat increment of feeding, also called specific dynamic action (Rubner 1902; Maynard and Loosli 1969) added at least partially to an increased level of time spent at *T*_b_ > 40 °C/days in fall, especially in mast years. To disentangle these effects, especially in free-living species, further research is needed.

The same is true for possible effects of body mass on locomotor activity and *T*_b_. In the present study, we were, unfortunately, not able to regularly record body mass data throughout the active season in free-ranging dormice. However, we observed high maximum *T*_b_’s (>40 °C) during different times of the active season when the animals most likely had different body masses (Fig. 1). For example, *T*_b_’s were elevated both during mating and prior to hibernation, whereas body mass is known to be low during mating and high prior to hibernation (Lebl et al. 2010). From first principles, we would expect a higher workload with increased body mass, which will result in an increased heat production during activity. On the other hand, increases in body mass also lead to an increased total heat capacity. Thus, elevated muscular work and thermogenesis in animals with higher body mass may not result in a detectable overall increase in *T*_b_. Accordingly, there is also no positive allometric relationship between body mass and *T*_b,_ neither among rodents nor among mammals in general (Clarke and Rothery 2008).

Interestingly, the highest periods of time spent at *T*_b_ > 40 °C/days were observed in males late in the active season in a mast-failure year (8 h/night). The fact that dormice hibernate in underground burrows might be important in this context. Digging is a costly locomotor activity and can increase MR up to 5.8 times BMR (reviewed in Karasov 1992). We cannot rule out that, towards the end of the active season, at least part of the phases with slight hyperthermia (hours spent at *T*_b_ > 40 °C/days) were related to digging of hibernacula. Since adult females show slightly higher site fidelity than adult males (J. Cornils pers. comm.), males might be forced more often to dig new hibernacula, while females may use the same hibernacula several times. This is an interesting question for further research, since nearly nothing is known about hibernacula in dormice (but see Bieber and Ruf 2009; Müller 1988).

### T_b_ and pregnancy in dormice

Females reduced the time spent at *T*_b_ > 40 °C/days during weeks 28–30 (minimum in week 29, 15–21 July). The estimated age of litters found in nest-boxes indicated that the late phase of pregnancy fell within this time frame. In laboratory mice, a significant decrease in activity could be observed during pregnancy towards parturition (Gamo et al. 2013). Since we observed in our free-living reproductive females a constant minimum *T*_b_ in weeks 28–30, we conclude that the decreased time spent at *T*_b_ > 40 °C/days (Fig. 2a) and the decreased maximum *T*_b_ (Fig. 4) is also related to a decrease in locomotor activity of pregnant dormice. Since the females may gain weight during pregnancy (litter weight at birth up to 26 g, Bieber and Ruf 2004), which is costly to carry around in an arboreal species, a decrease in activity seems plausible.

The date of parturition, however, matched with an increase in mean *T*_b_ (increase of 0.49 ± 0.04 °C Fig. 4). The associated high level of metabolism as well as the onset of milk production may lead to higher maternal *T*_b_ after birth (Speakman and Król 2010b). Thus, in dormice, like in several other species (reviewed in Williams et al. 2011), the date of parturition can be exactly determined by an increase in *T*_b_. However, in case of the Arctic ground squirrel the temperature abruptly increased by 1–1.5 °C after parturition (Williams et al. 2011), which is much higher than observed in dormice.

### Torpor in free-living dormice

The occurrence of short torpor bouts (<24 h) was strongly affected by the mast situation with much higher torpor frequencies in the mast-failure year (Fig. 5). Animals showed only few torpor bouts at the beginning of the active season (May) and during the intensive foraging period prior to hibernation. Females, for example, showed some bouts of torpor prior to gestation, when temperatures were still low (Fig. 4). For males, it is already known that only sexually quiescent animals use short bouts of torpor frequently, while sexually active males do not (Fietz et al. 2004). Thus, although torpor during the reproductive season has been observed in several hibernating species (reviewed in Geiser 2013), torpor and reproduction seem incompatible in dormice. High *T*_b_’s seem generally important for males to develop testis as shown, for example, in ground squirrels (Barnes et al. 1986; Michener 1992). In females, however, it seems that time constraints need to be considered in this context, since young are born late in the active season. A low *T*_b_ during pregnancy is known to slow down fetal development and would therefore further postpone the time of parturition (Racey and Swift 1981). A late date of birth, however, leads to a lower body mass in juveniles prior to hibernation, which might affect their survival probability (Pilastro et al. 1994).

Only immediately prior to the onset of hibernation, i.e., the first multiday torpor bout in fall, we observed the second phase of a strong increase in torpor frequency. We consider these bouts of torpor so-called “test drops” that occur at the onset of hibernation in many species (e.g., Sheriff et al. 2012; Strumwasser et al. 1967). However, the huge standard error of the mean (Fig. 5a) shows that some animals do not show these tests drops at all. Thus, they were not a prerequisite to enter hibernation, as is also the case in the Arctic ground squirrel (Sheriff et al. 2012).

Interestingly, animals were less active during phases with increased frequency of torpor (Fig. 6). Indeed these phases occurred during summer, when food availability (i.e., ripe fruits and seed) was low. In fall, on the other hand, dormice increased phases of time spent at *T*_b_ > 40 °C and reduced the use of torpor significantly. This strategy seems successful in terms of body mass gain, since body mass increases throughout the active season despite decreased food availability in mast-failure years (Bieber 1998; Schlund et al. 2002). A negative correlation between torpor frequency and nocturnal activity was also observed in a daily heterotherm, the Djungarian hamster (Ruf et al. 1991; Ruf and Heldmaier 1992). It has been suggested that the use of torpor has a negative impact on activity because the reduction of energy expenditure during torpor allows animals to minimize foraging activity (Ruf and Heldmaier 1992). This strategy should be particularly beneficial at times when foraging success is low, or when locomotor activity is risky or energetically costly.

### Effects of enclosure-housed conditions

We further compared the *T*_b_’s recorded in the field with data recorded from animals kept with ad libitum food supply in outdoor enclosures. Because of the restricted height of the enclosures (3.5 m) and the easily accessible food, we expected a lower locomotor activity, compared with field conditions. Our results confirm this hypothesis; however, even in our enclosures animals reached frequently *T*_b_’s above 40 °C (for on average 2.3 h/days) indicating that they are much more active than animals kept in small cages, in which hyperthermia was never observed (Wilz and Heldmaier 2000). This result indicates that data recorded under laboratory conditions, especially if animals are strongly restricted in their locomotor activity, should be interpreted with caution.

Interestingly, the study site did not affect the frequency of torpor. Thus, the control of short bouts of torpor is apparently not solely governed by food availability and/or climatic conditions. Similarly, the performance of summer dormancy in dormice is not related to energetic constraints or low *T*_a_’s (Bieber and Ruf 2009). The ability to escape predation, and therefore to increase survival probability, is an important factor that needs to be considered in this context (Bieber et al. 2014; Turbill et al. 2011; Geiser and Brigham 2012; Geiser and Turbill 2009; Bieber and Ruf 2009; Hoelzl et al. 2015). Although dormice do not vanish below ground during short bouts of torpor, it is likely that the correlated lower phases spent at *T*_b_ > 40 °C (i.e., physically active outside the nest) will lead to a decrease in predation risk, apart from energetic advantages. However, dormice seem to opt for strategies like summer dormancy and prolonged hibernation, if they can afford it (Hoelzl et al. 2015).

## Conclusion

The present study shows that *T*_b_ can serve as a valuable variable to obtain insights into the locomotor activity, as well as timing of reproduction in hibernators during the active season. Our data suggest that locomotor activity is strongly affected by environmental conditions, and that sexes respond differently to these changes. Hopefully our results will encourage future studies on *T*_b_ regulation and locomotor activity during the active season in hibernators. New devices, which allow measuring activity via accelerometers in combination with *T*_b_, should be very valuable in this context (Williams et al. 2016).
